# TRPV6 deficiency attenuates stress and corticosterone-mediated exacerbation of alcohol-induced gut barrier dysfunction and systemic inflammation

**DOI:** 10.3389/fimmu.2023.1093584

**Published:** 2023-01-31

**Authors:** Avtar S. Meena, Pradeep K. Shukla, Rupa Rao, Cherie Canelas, Joseph F. Pierre, RadhaKrishna Rao

**Affiliations:** ^1^ Department of Physiology, College of Medicine, University of Tennessee Health Science Center, Memphis, TN, United States; ^2^ Department of Pediatrics, College of Medicine, University of Tennessee Health Science Center, Memphis, TN, United States; ^3^ Memphis Veterans Affairs Medical Center, Memphis, TN, United States

**Keywords:** tight junction, inflammation, hepatitis, endotoxemia, stress, corticosterone

## Abstract

**Introduction:**

Chronic stress is co-morbid with alcohol use disorder that feedback on one another, thus impeding recovery from both disorders. Stress and the stress hormone corticosterone aggravate alcohol-induced intestinal permeability and liver damage. However, the mechanisms involved in compounding tissue injury by stress/corticosterone and alcohol are poorly defined. Here we explored the involvement of the TRPV6 channel in stress (or corticosterone) 3and alcohol-induced intestinal epithelial permeability, microbiota dysbiosis, and systemic inflammation.

**Methods:**

Chronic alcohol feeding was performed on adult wild-type and Trpv6-/- mice with or without corticosterone treatment or chronic restraint stress (CRS). The barrier function was determined by evaluating inulin permeability in vivo and assessing tight junction (TJ) and adherens junction (AJ) integrity by immunofluorescence microscopy. The gut microbiota composition was evaluated by 16S rRNA sequencing and metagenomic analyses. Systemic responses were assessed by evaluating endotoxemia, systemic inflammation, and liver damage.

**Results:**

Corticosterone and CRS disrupted TJ and AJ, increased intestinal mucosal permeability, and caused endotoxemia, systemic inflammation, and liver damage in wild-type but not Trpv6-/- mice. Corticosterone and CRS synergistically potentiated the alcohol-induced breakdown of intestinal epithelial junctions, mucosal barrier impairment, endotoxemia, systemic inflammation, and liver damage in wild-type but not Trpv6-/- mice. TRPV6 deficiency also blocked the effects of CRS and CRS-mediated potentiation of alcohol-induced dysbiosis of gut microbiota.

**Conclusions:**

These findings indicate an essential role of TRPV6 in stress, corticosterone, and alcohol-induced intestinal permeability, microbiota dysbiosis, endotoxemia, systemic inflammation, and liver injury. This study identifies TRPV6 as a potential therapeutic target for developing treatment strategies for stress and alcohol-associated comorbidity.

## Introduction

The intestinal mucosal barrier is critical for keeping the milieu interior separate from the luminal contents (1, 2). The epithelium serves as the primary barrier to the entry of luminal bacterial toxins, such as lipopolysaccharides (LPS), into the systemic circulation (3, 4). The tight junction (TJ) confers the epithelial barrier function, which blocks the diffusion of LPS from the gut lumen into the systemic circulation (5–8). Occludin, zonula occludens-1 (ZO-1), claudins, and cingulin are the major transmembrane proteins found in the tight junction (TJ). Interactions of transmembrane proteins with soluble proteins such as ZO-1 are crucial for maintaining epithelial barrier integrity (9–11). The TJ protein complex is connected to the underlying actin cytoskeleton belt (12). E-cadherin, β-catenin, and other proteins constitute the adherens junction (AJ) located beneath the TJ and indirectly regulate the TJ structural integrity (13, 14). A significant body of evidence demonstrates that ethanol (EtOH) and its toxic metabolite acetaldehyde disrupt intestinal epithelial TJ and AJ and cause barrier dysfunction by involving multiple signaling mechanisms, leading to endotoxemia and systemic inflammation (6, 15).

Alcohol abuse is a significant health concern for people across the world. It is well established that excessive use of alcohol over time leads to alcoholic liver damage (ALD); however, the precise mechanism involved in alcohol-associated liver injury is poorly defined. Systemic inflammation is the common denominator in the pathogenesis of alcohol use disorders (AUD) (16, 17). Alcohol causes inflammation in various organs, including the brain, intestines, pancreas, and liver. Gut barrier dysfunction and the consequent endotoxemia are crucial contributing factors in developing systemic inflammation in AUD (16, 18–20). Intestinal microbiota dysbiosis, associated with elevated LPS in the gut, is a crucial contributor to alcohol-associated endotoxemia. LPS is absorbed into the portal circulation, which targets and activates Kupffer cells and hepatocytes in the liver. LPS-activated liver cells secrete chemokines and cytokines that promote inflammation (21, 22). Once it has entered the systemic circulation, LPS causes systemic inflammation, thus affecting multiple organs. As a result, heavy drinkers almost always develop fatty liver; however, only about 35 percent of alcoholics develop hepatitis, and only 10-20 percent of alcoholics develop cirrhosis (23). Therefore, “second-hit” or “multiple hits” models of ALD pathogenesis have been proposed.

A recent study suggested that stress and corticosterone may contribute to alcohol-related tissue damage, and stress is likely a second hit in the pathogenesis of alcohol-induced tissue injury (20). In animal models and patients with irritable bowel syndrome, chronic stress disrupts gastrointestinal motility, increases visceral pain perception, and impairs intestinal barrier function (24, 25). Chronic stress alters gut-brain axis regulation and promotes pro-inflammatory cytokine production potentially by elevating the plasma glucocorticoids (20, 26), resulting in the alteration of gastrointestinal, cognitive, and emotional pathways. Stress stimulates the hypothalamic-pituitary-adrenal (HPA) axis with a sustained increase in plasma glucocorticoids, which plays an essential role in the pathophysiology of stress-related disorders (27, 28). Cortisol (corticosterone in rodents) is the primary stress hormone that plays a crucial role in stress responses. Elevated cortisol is associated with multiple health problems (29). Corticosterone modulates chronic psychological stress-induced impairment of colonic epithelial barrier function in a region-specific manner by decreasing the expression of epithelial tight junction proteins and increasing epithelial permeability (20). Chronic stress is co-morbid with AUD (30–32); chronic stress and alcohol abuse feedback into each other, worsening clinical symptoms of both disorders. Evidence suggests alcohol abuse disrupts the HPA axis and increases plasma glucocorticoids (33–35). Chronic stress and alcohol consumption increases plasma corticosterone levels in animal models (19, 36, 37). A previous study demonstrated that chronic stress and corticosterone exacerbate alcohol-induced pathophysiology in the Gut-Liver-Brain axis (20). Corticosterone-mediated intestinal epithelium sensitization to alcohol-induced TJ disruption and gut microbiota dysbiosis may define stress-induced exacerbation of alcohol-associated tissue damage (20), suggesting that persistent stress may have an exacerbating role in the pathogenesis of alcohol-related illnesses. Therefore, it is essential to define the mechanisms of the pathophysiology of ALD to identify the early targets of alcohol and corticosterone-induced gut barrier dysfunction.

The precise mechanisms involved in stress, corticosterone, or alcohol-induced intestinal epithelial TJ and AJ are poorly defined. A recent study has demonstrated that the transient receptor potential vanilloid 6 (TRPV6) channel plays a crucial role in alcohol-induced TJ breakdown, barrier impairment, endotoxemia, and increased inflammation. TRPV6 is a calcium-permeable ion channel expressed on the apical membrane of the intestinal epithelial cells in humans, mice, and rats (36, 38, 39).. Our recent study demonstrated that EtOH and acetaldehyde activate TRPV6 in the intestinal epithelium, increase intracellular calcium, and disrupt TJ (18). In addition, TRPV6-deficient mice are resistant to alcohol-induced barrier dysfunction, systemic inflammation, liver injury, and neuronal injury. Our research identifies TRPV6 as a potential early candidate in the pathophysiology of alcohol-associated diseases, raising the question of whether TRPV6 plays a role in the effect of corticosterone on basal and alcohol-induced gut permeability, endotoxemia, systemic inflammation, and liver damage. Therefore, we investigated the involvement of the TRPV6 channel in stress- and corticosterone-induced potentiation of intestinal permeability, endotoxemia, and systemic inflammation.

## Materials and methods

### Chemicals and antibodies

Regular Lieber-DeCarli diet and maltodextrin were obtained from Dyets Inc (Cat# 710260; Bethlehem, PA, USA) and Bioserv (Flemington, NJ, USA), respectively. Corticosterone was procured from Sigma-Aldrich (St Louis, MO, USA). Antibodies for occludin and ZO-1 were procured from Thermo Fisher Scientific (Waltham, MA, USA). Antibodies against E-cadherin and β-catenin were procured from BD Biosciences (San Jose, CA, USA). Secondary antibodies conjugated with AlexaFluor-488 or Cy3 were procured from Molecular Probes (Eugene, OR, USA). All other antibodies or chemicals were procured from either Thermo Fisher Scientific or Sigma-Aldrich (St Louis, MO, USA).

### Animals and diets

Mouse strain deficient in the TRPV6 gene was a generous gift from Prof. Matthias A. Hediger, University of Bern, Switzerland), and acquired through Dr. Sylvia Christakos (Rutgers New Jersey Medical School, Newark, NJ, USA). TRPV6 mice have been backcrossed to C57BL/6 mice for more than 20 generations. The wild-type mice were derived from the same colony and are littermates. The Institutional Animal Care and Use Committee at UTHSC have approved all protocols and guidelines for animal experiments described in this study. Animals (two per cage) were housed in a 12:12 h light: dark cycle conditions in our institutional core facility. Animals had unrestricted access to standard laboratory chow and water before the experiments.

### EtOH feeding and stress

*Chronic EtOH feeding*: Adult *Trpv6^-/-^* and its corresponding wild-type mice (age: 8–10 weeks, male and female) were fed Lieber–DeCarli liquid diet containing varying concentrations of EtOH (0% for two days, 1% for two days, 2% for two days, 4% for one week, 5% for one week, and 6% for one week) for four weeks as described previously (21, 40). In control groups, EtOH was substituted with isocaloric maltodextrin. Diet consumption was recorded daily, and body weight measurements were documented twice a week. The pair-fed and EtOH-fed mice were subjected to one of the two kinds of stressors.

#### Chronic stress

Mice were subjected to chronic restraint stress (CRS) for two hours daily during EtOH feeding, as previously reported (20). Briefly, the adult *Trpv6^-/-^* and wild-type mice were physically restrained for two hours per day using the TV150 restrainer (Braintree Scientific, Braintree, MA) (from 9–11 AM). Parallelly, non-stressed mice were restricted to food and drink for two hours daily without restraint (Sham treatment). The animals were transferred to a liquid diet five days after CRS was started; CRS was continued until the end of the experiment.

#### Corticosterone administration

Corticosterone was injected at a dose of 25 mg/kg per day (s.c.) in some groups of pair-fed and EtOH-fed mice for four weeks, as reported before (37). The vehicle was administered to control mice. Animals were always housed in pairs to help maintain body temperature and avoid social isolation.

### Gut permeability *in vivo*

The mucosal permeability of the colon and ileum was assessed as described previously (20). At the end of the study, mice were restrained and injected with FITC-inulin (100 mg/ml solution: 2 μl/g body weight) through their tail veins. One hour following infusion, blood samples were drawn by cardiac puncture under isoflurane anesthesia to prepare plasma, followed by euthanizing mice by cervical dislocation. The 0.9% saline solution was used for flushing the intestinal segments’ luminal contents. Fluorescence in plasma and luminal flushing samples was measured using a fluorescence plate reader. Fluorescence in the luminal flushing was correlated with the plasma fluorescence; results are presented as percentages of the inulin load to mice. There are multiple ways to measure intestinal permeability in mice *in vivo*. Each of them has advantages and disadvantages. Luminal-to-vascular and vascular-to-luminal permeability measurements show no differences as permeability through leaky tight junction has no directionality. The vascular-to-luminal permeability measurements involve the minor manipulation of the intestine, and we have consistently shown an excellent correlation between permeability measured by this method and TJ disruption by confocal microscopy in corresponding mice in previous studies.

### Immunofluorescence microscopy

Colon cryosections (10 μm) were fixed in an acetone and EtOH (1:1) mixture at −20°C for 2 minutes and then rehydrated in phosphate-buffered saline (PBS). Fifteen minutes of permeabilization with 0.2% Triton X-100 in PBS was followed by blocking with 4% nonfat milk in Triton-Tris buffer. Next, a primary antibody specific for TJ and AJ (1:100 dilution) was added and incubated for one hour, followed by incubation with secondary antibodies for an additional hour. After incubations with antibodies, the section was incubated with Hoechst 33342 for 10 minutes, and fluorescence images were captured using a Zeiss 710 confocal microscope (Carl Zeiss Microscopy, Jena, Germany). Images were stacked and processed using Image J software (NIH, Bethesda, MD) and Adobe Photoshop (Adobe Systems Inc., San Jose, CA).

### Quantitative RT-PCR

The analysis of particular mRNA was performed by RT-PCR as described previously (37). cDNAs were synthesized from 1.5 μg of RNA using the ThermoScript RT-PCR instrument for first-strand synthesis (Invitrogen). Quantitative PCR (qPCR) reactions were performed using a cDNA mix with primers in a final volume of 25 μl in an Applied Biosystems Quant Studio 6-Flex Real-Time PCR instrument (Norwalk, CT, USA). The parameter of the cycle was as follows: 50°C for two minutes, one step of denaturation at 95°C for ten minutes, and 40 cycles of denaturation at 95°C for ten seconds, followed by annealing and elongation at 60°C. The ΔΔCt analysis normalized each transcript’s relative expression level to that of GAPDH. Table S1 of the Supplemental Information presents the primer sequences used for qPCR.

### Microbiome analyses

The microbiome of colonic flushing was analyzed using 16S rRNA sequencing and metagenomic analysis, as described in the previous study (20).

#### Microbial DNA isolation

Fecal samples from mice were homogenized in extraction buffer [50mM Tris (pH 7.4), 0.1 M EDTA (pH 8.0), 0.4 M NaCl, 0.5% SDS] with 20 μL of 20 mg/ml proteinase K, Zirconia/silica beads of 0.1 mm diameter (BioSpec Products, Bartlesville, OK, USA). A Mini-Beadbeater-8 cell disrupter (BioSpec Products) was used to break up microbial cells in the extraction tubes (2 x 1 min). The samples were mixed for 16 hours at 55°C, extracted using phenol, chloroform, and isoamyl alcohol, precipitated in 70% EtOH, and centrifuged at 10,000 x g for 10 min. Before sequencing, genomic DNA extract was reconstituted in nuclease-free water and preserved at 80°C.

#### PCR based on 16S rRNA, library preparation using the Illumina sequencer, and analytical methods

Primers with Illumina 3’ adapter sequences and unique 12-bp barcodes were used for the amplicon sequencing of the 16S rRNA V4-V5 region (338F: 5’-GTGCCAGCMGCCGGTAA-3’ and806R: 5’-GGACTACHVGGGTWTCTAAT-3’). Sequences were performed at NovoGene using an Illumina MiSeq D.N.A. platform and processed using Quantitative Insights Into Microbial Ecology (QIIME). Using an open reference operational taxonomic unit (OTUs), OTUs with 97% sequence identity were identified and picked against the green genes database. Sequences were rarified to an equivalent sampling depth of 5,000 reads for each sample, and OTUs were quality filtered based on the standard criteria defined in the open-reference OTU function in QIIME. The specific and representative sequences were aligned through PyNAST, the taxonomy of the sequence was assigned using the RDP Classifier, and a phylogenetic tree was created using FastTree. Bray Curtis measurements given as Principal Coordinate Analysis (PCoA) graphs represent Beta Diversity. Calypso was applied to generate Spearman heat maps and Redundancy Analysis on OTU tables (41). As shown in the figure legends, PERMANOVA was designed to estimate the relevance of beta diversity. The Shannon index was implemented to examine alpha diversity at the genus level. Taxa tables obtained in QIIME were further examined across samples using MicrobiomeAnalyst to conduct Pattern Search and Network Analysis (42).

### Plasma endotoxin analysis

Using a Pierce LAL Chromogenic Endotoxin Quantitation Kit (Thermo Scientific), plasma endotoxin concentration was measured as described in our previous studies (20, 37).

### Plasma cytokine assay

Plasma cytokine and chemokine levels were evaluated using a Duoset ELISA kit (R&D system, Minneapolis, MN) according to the manufacturer’s instructions. Briefly, 50 μL of plasma was incubated overnight on microplates treated with capture antibody, followed by a 2-hour incubation with detection antibody. The sample was then probed for 20 minutes with streptavidin conjugated with horseradish peroxidase and substrate solution. After terminating the reaction with a stop-solution, the absorbance was determined at 450 nm and normalized for wavelength at 570 nm.

### Liver histopathology

Liver tissues were fixed in 10% buffered formalin, and 8 μm-thick paraffin-embedded slices were stained with hematoxylin and eosin (H&E) as detailed previously (20, 37). Stained sections were imaged using a Nikon 80Ti microscope (Nikon Instruments, Inc., Melville, NY) with a 10x objective lens and a color camera.

### Plasma transaminase assay

The activities of plasma aspartate transaminase (AST) and alanine transaminase (ALT) were determined using a colorimetric assay using ALT (Cayman, Cat#700260) and AST (Cayman, Cat#7012640) assay kits, respectively, according to the vendor’s instructions.

### Triglyceride assay

The triglyceride content in the liver was assessed by the GPO method using the previously described assay kit from Pointe Scientific Inc. (Canton, MI) (20, 37). Briefly, Tissue lipids were extracted by treating the sample in 3 M potassium hydroxide (in 65% EtOH) at 70°C for one hour and at room temperature overnight. Enzymatic hydrolysis of triglycerides into glycerol and free fatty acids was followed by spectrophotometric determination of glycerol at 540 nm. The hepatic triglyceride values were represented as mg triglyceride/g liver tissue.

### Statistical analyses

All data are presented as Mean ± SEM. ANOVA was used to examine the differences between several groups (Prism 6.0). Tukey’s t-test was employed to determine the statistical significance between different testing sets and the respective control. The statistical significance was determined with 95% confidence.

The values are shown as the Mean ± standard error of the mean (SEM) (n =4-6); * p <0.05, ** p <0.01, *** p <0.005, and **** p <0.001 signify a significant difference between the groups that are specified; ns denotes that the difference is not significant.

## Results

### TRPV6 plays a role in the corticosterone-mediated potentiation of alcohol-induced gut mucosal injury

**Figure 1:**
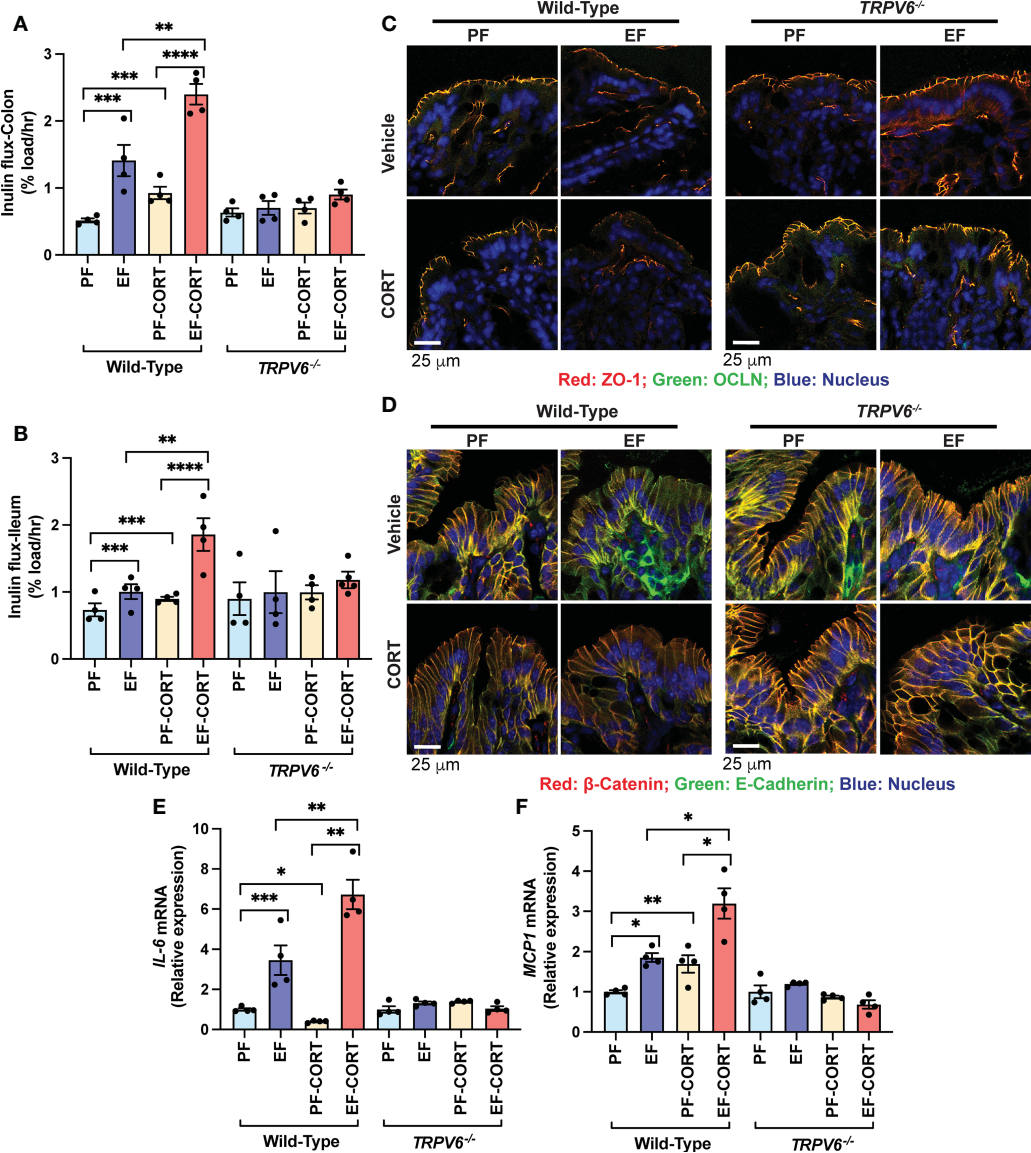
TRPV6 deficiency attenuates corticosterone and alcohol-induced gut permeability, epithelial junctional disruption, and mucosal inflammatory response. For four weeks, adult wild-type and *Trpv6^-/-^* mice were fed a liquid diet supplemented with EtOH (EF) or isocaloric maltodextrin (PF). In addition, some animal groups received daily doses of corticosterone (CORT). Other groups of animals were administered with the vehicle. **(A, B)** The vascular-to-luminal flux of FITC-inulin was measured *in vivo* to assess the mucosal permeability of the colon **(A)** and ileum **(B)**. **(C)** Immunofluorescence labeling of colon cryosections for occludin and ZO-1 (occludin: green; ZO-1: red; nucleus: blue) and confocal imaging were used to evaluate TJ integrity. **(D)** The integrity of adherens junctions were evaluated by labeling colon sections with E-cadherin and β-catenin (E-cadherin: green; β-catenin: red; nucleus: blue). **(E,F)** RNA from colonic mucosa was examined by RT-qPCR for *IL-6* **(E)** and *MCP1* **(F)** mRNA. Values are Mean ± SEM (n = 4); *p <0.05, **p <0.01, ***p <0.005, and ****p <0.001 for significant difference between the indicated groups; ns, not significant.

Corticosterone disrupts the intestinal mucosal barrier and potentiates EtOH-induced barrier dysfunction (20). EtOH and AA disrupt TJ in Caco-2 cell monolayers by an intracellular calcium-dependent mechanism (15). TRPV6 is essential for alcohol-induced calcium influx in the intestinal mucosa, TJ breakdown, endotoxemia, systemic inflammation, and liver damage (18). To determine whether TRPV6 plays a role in corticosterone-induced intestinal mucosal injury and corticosterone-induced potentiation of alcohol effects on the gut and liver, we fed wild-type and *Trpv6^-/-^* mice EtOH for four weeks. In wild-type mice, EtOH-feeding enhanced mucosal permeability to FITC-inulin (6 kDa) in the colon (**Figure 1A**) and ileum (**Figure 1B**). Corticosterone, by itself, also significantly elevated the colonic mucosal permeability, and it synergistically potentiated the EtOH-induced increase in colonic mucosal permeability. *Trpv6^-/-^* mice resisted corticosterone-induced intestinal mucosal permeability and corticosterone-mediated promotion of alcohol-induced mucosal permeability. An increase in mucosal permeability suggested that corticosterone may disrupt TJ and AJ. Therefore, cryosections of the distal colon were stained for TJ and AJ proteins. The presence of occludin and ZO-1 at the intercellular junctions of the intestinal epithelium of pair-fed wild-type and *Trpv6^-/-^* mice indicates the high integrity of TJ in pair-fed animals. Corticosterone alone elicited a modest displacement of occludin and ZO-1 from epithelium in wild-type mice. The TJ disruption was more pronounced when EtOH feeding was combined with corticosterone administration than EtOH or corticosterone alone (**Figure 1C**). Densitometry of fluorescence at the epithelial junctions confirmed a significant decrease in the junctional distribution of ZO-1 in EtOH-fed mice, which was more severe when combined with corticosterone treatment (**Supplementary Figure S2A**). Corticosterone also reduced E-cadherin and β-catenin fluorescence in the colonic epithelium of wild-type mice but not in *Trpv6^-/-^* mice (**Figure 1D**); confirmed by densitometry of β-catenin at the epithelial junctions (**Supplementary Figure S2B**). The impact of EtOH and corticosterone on the redistribution of E-cadherin and β-catenin appears to be more severe in wild-type mice; such an effect of EtOH and corticosterone was absent in the *Trpv6^-/-^* mice.

We measured *IL-6* and *MCP1* mRNA in the colonic mucosa as an indicator of mucosal inflammatory response. EtOH significantly increased *IL-6* (**Figure 1E**) and *MCP1* (**Figure 1F**) mRNA. Corticosterone increased *MCP1* mRNA and potentiated EtOH-induced increases in *MCP1* mRNA in the wild-type mice but not in *Trpv6^-/-^* mice (**Figure 1E**). Corticosterone reduced *IL-6* mRNA, but it synergistically potentiated the EtOH-induced increase in *IL-6* mRNA (**Fig 1F**). The effects of corticosterone and EtOH-induced colonic inflammatory response were absent in *Trpv6^-/-^* mice. These data suggest that corticosterone exacerbates the alcohol-induced intestinal epithelial barrier dysfunction and mucosal inflammatory response by a TRPV6-dependent mechanism.

The diet intake was similar in all groups except the corticosterone treatment groups (**Supplementary Figure S1A**). Similar diet intake in EtOH-fed groups (with or without corticosterone) in wild-type and *Trpv6^-/-^* mice indicate that the EtOH consumption was similar in these strains. Neither EtOH nor corticosterone treatment altered the body weight changes during the experiment (**Supplementary Figure S1B-E**); corticosterone treatment alone tends to increase the body weight; however, the differences were not statistically significant. In addition, the colon length was considerably shortened by EtOH feeding in wild-type mice, whereas it was slightly increased when EtOH was combined with corticosterone treatment (**Supplementary Figure S1F**); such differences in colon length were absent in *Trpv6^-/-^* mice.

### TRPV6 deficiency attenuates corticosterone and alcohol-induced endotoxemia and systemic inflammation

**Figure 2:**
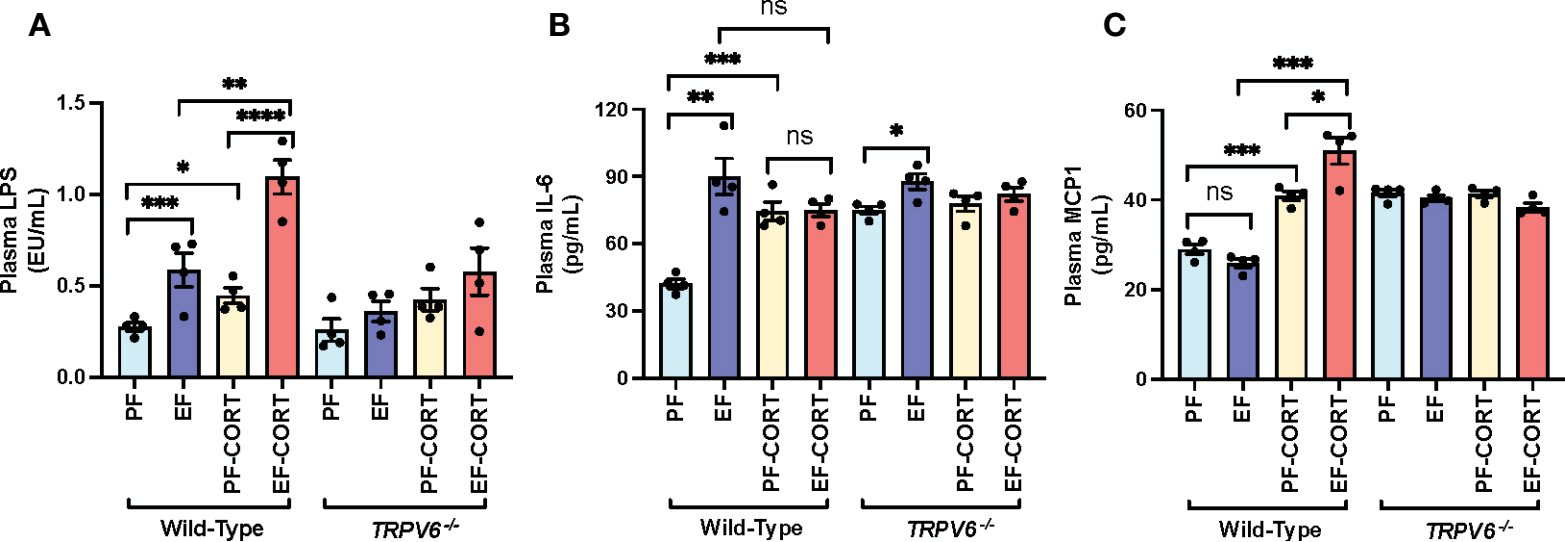
Corticosterone- and alcohol-induced endotoxemia and systemic inflammation are absent in TRPV6 deficient mice. For four weeks, adult wild-type and *Trpv6^-/-^* mice were fed a liquid diet supplemented with EtOH (EF) or isocaloric maltodextrin (PF). In addition, some animal groups received daily doses of corticosterone (CORT). Other groups of animals were administered with the vehicle. **(A)** Endotoxemia was evaluated by monitoring LPS levels in plasma. **(B, C)**The levels of IL-6 **(B)** and MCP1 **(C)** in blood plasma were evaluated by ELISA. Values in graphs are Mean ± SEM (n = 4); *p <0.05, **p <0.01, and ***p <0.005 for significant difference between the indicated groups; ns, not significant.

Epithelial TJ disruption allows LPS absorption from the intestinal lumen into the portal and systemic circulation, leading to elevated plasma LPS, a condition referred to as endotoxemia (6). Therefore, we assessed the plasma LPS as an indicator of endotoxemia. Results show that EtOH and corticosterone significantly increased plasma LPS in wild-type mice (**Figure 2A**). The combined EtOH and corticosterone treatments induced a synergistic elevation of plasma LPS, the effect more remarkable than the sum of EtOH and corticosterone-alone effects. This effect of corticosterone and corticosterone-mediated potentiation of EtOH effects on plasma LPS were absent in *Trpv6^-/-^* mice. LPS in the circulation stimulates immune cells in multiple organs resulting in a systemic inflammatory response. Therefore, we evaluated the plasma concentration of representative cytokines as the indicator of systemic inflammation. EtOH or corticosterone alone significantly elevated plasma IL-6 in the wild-type mice. Plasma IL-6 in pair-fed *Trpv6^-/-^* mice were slightly, but significantly, more significant than that in pair-fed wild-type mice; however, neither EtOH nor corticosterone elevated plasma IL-6 in *Trpv6^-/-^* mice (**Figure 2B**). Corticosterone treatment elevated plasma MCP1, whereas EtOH feeding caused a slight decrease (**Figure 2C**). However, EtOH and corticosterone together produced a synergistic elevation of plasma MCP1. Plasma MCP1 in pair-fed *Trpv6^-/-^* mice was considerably more significant than in pair-fed wild-type mice. However, EtOH, corticosterone, or EtOH + corticosterone failed to increase plasma MCP1 levels (**Figure 2C**). These findings suggest that TRPV6 is required for corticosterone- and corticosterone-mediated potentiation of EtOH-induced endotoxemia and systemic inflammatory response.

### Corticosterone-induced exacerbation of alcohol-induced liver damage is minimal in TRPV6-deficient mice

**Figure 3:**
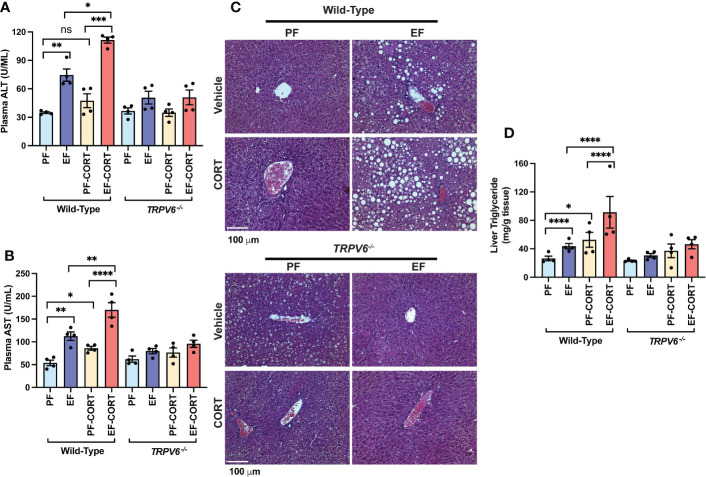
TRPV6 deficiency attenuates corticosterone and alcohol-induced liver damage. For four weeks, adult wild-type and *Trpv6^-/-^* mice were fed a liquid diet supplemented with EtOH (EF) or isocaloric maltodextrin (PF). In addition, some animal groups received daily doses of corticosterone (CORT). Other groups of animals were administered with the vehicle. **(A, B)** Alanine aminotransferase **(A)** and aspartate aminotransferase **(B)** activities in blood plasma were examined. **(C)** Bright field microscopy and H&E stains were used to perform liver histology. **(D)** Steatosis was determined by evaluating triglyceride levels in the liver. Values in graphs are Mean ± SEM (n = 4); *p <0.05, **p <0.01, ****p <0.005, and ****p <0.001 for significant difference between the indicated groups; ns, not significant.

Endotoxemia and intestinal barrier disruption play essential roles in the pathophysiology of alcohol-related liver damage. The liver is the initial organ that receives portal LPS. Therefore, we assessed liver damage in wild-type and *Trpv6^-/-^* mice. In wild-type mice, the plasma levels of liver injury markers ALT (**Figure 3A**) and AST (**Figure 3B**) were elevated by EtOH and corticosterone. EtOH and corticosterone in combination caused synergistically higher levels of plasma ALT and AST in wild-type mice. These effects of EtOH and corticosterone (alone or together) on plasma ALT and AST were minimal in *Trpv6^-/-^* mice. The changes in the plasma liver markers were associated with parallel changes in liver histopathology. In wild-type mice, EtOH feeding revealed significant liver histopathological changes characterized by white droplets of varying sizes, representing the fat deposits predominantly in the centrilobular zone, a condition referred to as fatty liver (**Figure 3C**). In wild-type mice, corticosterone alone did not cause gross histopathological changes, but it increased the severity of EtOH-induced histopathological changes in wild-type mice. These effects of EtOH were absent in *Trpv6^-/-^* mice in the absence or presence of corticosterone. EtOH-induced fatty liver was confirmed by measuring the liver triglyceride content. In wild-type mice, EtOH and corticosterone alone significantly increased liver triglyceride; however, the increase was much more significant when EtOH feeding was paired with corticosterone treatment (**Figure 3D**). These effects were minimal and non-significant in *Trpv6^-/-^* mice. These findings suggest that TRPV6 is necessary for EtOH- and corticosterone-mediated potentiation of EtOH-induced liver injury. EtOH-feeding significantly reduced liver weights relative to body weights in the absence of corticosterone in wild-type mice (**Supplementary Figure S1G**); however, liver weight was increased by EtOH in the presence of corticosterone. These changes in liver weights were absent in *Trpv6^-/-^* mice.

### TRPV6 deficient mice resist chronic stress and stress-mediated potentiation of alcohol-induced disruption of epithelial junctions, mucosal permeability, and inflammatory response in the colon

**Figure 4:**
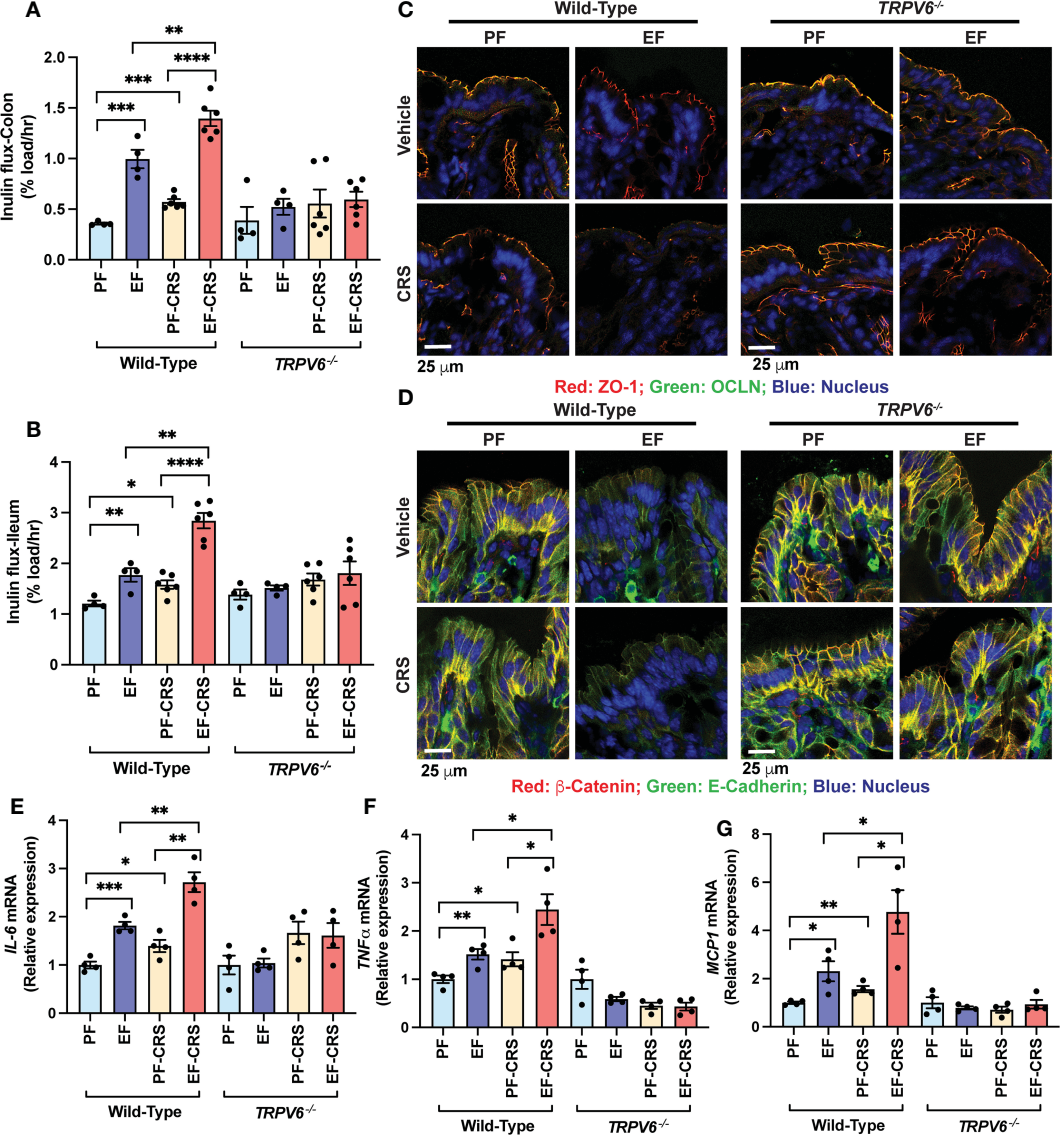
TRPV6 deficiency attenuates stress and alcohol-induced gut permeability, epithelial junctional disruption, and mucosal inflammatory response. For four weeks, adult wild-type and *Trpv6^-/-^* mice were fed a liquid diet supplemented with EtOH (EF) or isocaloric maltodextrin (PF). Some animal groups were subjected to daily two-hour chronic restraint stress (CRS). Non-stressed groups were prohibited from food and drink for two hours daily (Sham). **(A, B)** By evaluating the vascular-to-luminal flux of FITC-inulin *in vivo*, the mucosal permeability of the colon **(A)** and ileum **(B)** was assessed. **(C)** Immunofluorescence labeling of colon cryosections for occludin and ZO-1 (occludin: green; ZO-1: red; nucleus: blue) and confocal imaging were utilized to evaluate the integrity of tight junctions. **(D)** Colon sections were stained for E-cadherin and β-catenin to determine the integrity of adherens junctions (E-cadherin: green; β-catenin: red; nucleus: blue). **(E–G)** RNA extracted from colonic mucosa was subjected to RT–qPCR for the detection of mRNA specific for *IL-6* **(E)**, *TNF-α***(F)**, and *MCP1* **(G)**. Values are Mean ± SEM (n = 4); *p <0.05, **p <0.01, ***p <0.005, and ****p <0.001 for significant difference between the indicated groups; ns, not significant.

To investigate endogenous corticosterone’s effect, we examined chronic restraint stress (CRS) on gut and liver injury and potentiation of alcohol-induced gut mucosal barrier dysfunction, endotoxemia, and liver injury in wild-type and *Trpv6^-/-^* mice. In wild-type mice, EtOH feeding significantly elevated mucosal inulin permeability in the colon (**Figure 4A**) and ileum (**Figure 4B**). CRS also elevated the mucosal permeability to inulin in the ileum and colon. The combination of EtOH feeding and CRS resulted in a much more severe increase in mucosal permeability in wild-type mice; the effect was more significant than the sum of the effects of EtOH and CRS by themselves (**Figures 4A, B**). The effects of EtOH and CRS, individually or combined, on mucosal permeability were absent in *Trpv6^-/-^* mice. Increased mucosal permeability suggested that EtOH and CRS disrupt TJ and AJ. Results indicate that TJ proteins (occludin and ZO-1) are co-localized at the intercellular junction of colonic epithelium in both pair-fed wild-type and *Trpv6^-/-^* mice. In the wild-type mice, EtOH or CRS alone induced a moderate junctional redistribution of TJ from the epithelial junctions; these effects were much more severe in the combined EtOH and CRS treatments compared to EtOH or CRS group (**Figure 4C**). Densitometry of fluorescence at the epithelial junctions confirmed a significant decrease in the junctional distribution of ZO-1 in EtOH-fed mice (**Supplementary Figure S4A**). EtOH and CRS also reduced AJ proteins (E-cadherin and β-catenin) fluorescence in the colonic epithelium of wild-type mice but not in *Trpv6^-/-^* mice (**Figure 4D**). The effects of EtOH and CRS combination on the E-Cadherin and β-catenin distribution appears to be more severe in wild-type mice, and such an effect of EtOH and CRS was absent in *Trpv6^-/-^* mice; confirmed by densitometry of β-catenin at the epithelial junctions (**Supplementary Figure S4B**).

We measured *IL-6*, *TNFα*, and *MCP1* mRNA in the colonic mucosa as the indicators of mucosal inflammatory response. EtOH increased *IL-6* (**Figure 4E**), *TNFα* (**Figure 4F**), and *MCP1* mRNA (**Figure 4G**). CRS also increased *IL-6*, *TNFα*, and *MCP1* mRNA and potentiated EtOH-induced increases in cytokine mRNA expression in wild-type mice but not in *Trpv6^-/-^* mice. These data suggest that CRS exacerbates the alcohol-induced intestinal epithelial barrier dysfunction and mucosal inflammatory response by a TRPV6-dependent mechanism. The diet intake was similar in all groups, except for a slightly higher diet intake in EtOH-fed *Trpv6^-/-^* mice compared to that in EtOH-fed wild-type mice (**Supplementary Figure S3A**). EtOH-feeding significantly reduced body weight gain in wild-type mice during treatments; this effect was independent of CRS treatment (**Supplementary Figure S3B–E**). EtOH-induced weight loss was absent in *Trpv6^-/-^* mice. EtOH-supplementation dramatically increased the colon length in wild-type mice, irrespective of CRS (**Supplementary Figure S3F**); EtOH-induced colon length increases were absent in *Trpv6^-/-^* mice.

### TRPV6 deficiency attenuates stress and alcohol-induced endotoxemia, and systemic inflammation

**Figure 5:**
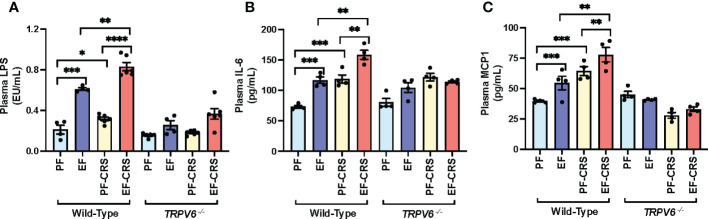
Stress and alcohol-induced endotoxemia and systemic inflammation are absent in TRPV6 deficient mice. For four weeks, adult wild-type and *Trpv6^-/-^* mice were fed a liquid diet supplemented with EtOH (EF) or isocaloric maltodextrin (PF). Some animal groups were subjected to daily two-hour chronic restraint stress (CRS). Non-stressed groups prohibited food and drink for two hours daily (Sham). **(A)** Plasma LPS levels were measured in order to evaluate endotoxemia. **(B, C)** Using an ELISA, plasma levels of IL-6 **(B)** and MCP1 **(C)** were determined. Values in graphs are mean ± SEM (n = 4); *p <0.05, **p <0.01, and ***p <0.005 for significant difference between the indicated groups; ns, not significant.

Disrupted epithelial TJ permits LPS to enter the mucosa from the intestinal lumen, causing endotoxemia and triggering a systemic inflammatory response. Results show that EtOH and CRS alone significantly increased plasma LPS in wild-type mice (**Figure 5A**). The combined EtOH-feeding and CRS induced a synergistic elevation of plasma LPS, the effect more significant than the sum of EtOH and CRS effects. These effects of CRS and CRS-mediated potentiation of EtOH-induced elevation of plasma LPS were absent in *Trpv6^-/-^* mice. EtOH and CRS alone significantly elevated plasma *IL-6* (**Figure 5B**) and *MCP1* (**Figure 5C**) in the wild-type mice; the cytokine levels were significantly higher when EtOH and CRS were combined. The effects of EtOH and CRS on plasma cytokines, alone or in combination, were minimal in *Trpv6^-/-^* mice. These data suggest that TRPV6 is required for CRS and CRS-mediated potentiation of EtOH-induced endotoxemia and systemic inflammation.

### TRPV6 is necessary for alcohol- and stress-induced liver damage

**Figure 6:**
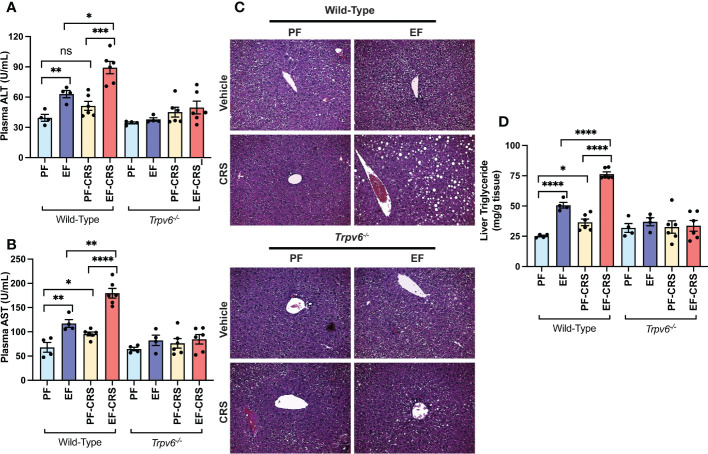
TRPV6 deficiency attenuates stress and alcohol-induced liver damage. For four weeks, adult wild-type and *Trpv6^-/-^* mice were fed a liquid diet supplemented with EtOH (EF) or isocaloric maltodextrin (PF). Some animal groups were subjected to daily two-hour chronic restraint stress (CRS). Non-stressed groups were prohibited from food and drink for two hours daily (Sham). **(A, B)** Plasma was examined to determine the levels of alanine aminotransferase **(A)** and aspartate aminotransferase **(B)** activities. **(C)** H&E staining and bright field microscopy were used to perform liver histopathology. **(D)** The level of steatosis was determined by measuring the level of triglycerides in the liver. Values in graphs are Mean ± SEM (n = 4); *p <0.05, **p <0.01, ****p <0.005, and ****p <0.001 for significant difference between the indicated groups; ns, not significant.

The prior investigation established that CRS exacerbated EtOH-induced liver damage in murine models (37). Here, we confirm the CRS effect on liver damage in wild-type mice. The plasma ALT (**Figure 6A**) and AST (**Figure 6B**) activities were elevated by EtOH-feeding and CRS alone in wild-type mice. EtOH and CRS, in combination, caused synergistically higher levels of plasma ALT and AST in wild-type mice. No significant effects of EtOH and CRS (alone or together) on plasma ALT and AST were recorded in *Trpv6^-/-^* mice. Histopathological analyses of the EtOH-fed wild-type liver showed significant pathologic changes characterized by varying-sized white droplets, representing the fat deposits predominantly in the centrilobular zone (**Figure 6C**). CRS alone did not cause gross histopathological changes, but it increased the severity of EtOH-induced histopathologic changes in wild-type mice. These effects of EtOH alone or combined with CRS were absent in *Trpv6^-/-^* mice. EtOH-induced fatty liver was confirmed by measuring the liver triglyceride content. In wild-type mice, EtOH and CRS alone significantly increased liver triglyceride; however, the increase was significantly greater when EtOH supplementation was combined with CRS (**Figure 6D**). These effects were minimal and non-significant in *Trpv6^-/-^* mice. These findings suggest that TRPV6 is necessary for EtOH- and CRS-mediated potentiation of EtOH-induced liver injury. EtOH-feeding significantly increased liver weights relative to body weights in wild-type mice, irrespective of CRS (**Supplementary Figure S3G**); EtOH-induced liver weight increases were absent in *Trpv6^-/-^* mice.

### TRPV6 deficiency attenuates stress and alcohol-induced dysbiosis of gut microbiota

**Figure 7:**
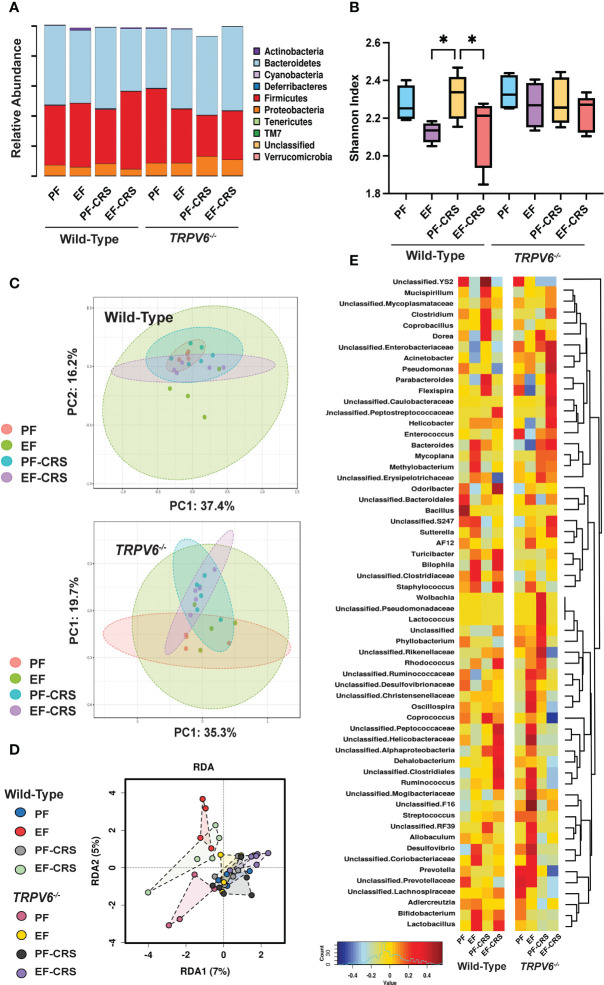
TRPV6 deficiency attenuates stress and alcohol-induced dysbiosis of gut microbiota. For four weeks, adult wild-type and *Trpv6^-/-^* mice were fed a liquid diet supplemented with EtOH (EF) or isocaloric maltodextrin (PF). Some animal groups were subjected to daily two-hour chronic restraint stress (CRS). Non-stressed groups were prohibited from food and drink for two hours daily (Sham). 16S rRNA sequencing and metagenomics were used to examine the bacterial diversity of colonic flushing. **(A)** Microbiota in terms of their relative abundance at the phylum level is displayed by experimental groups. **(B)** The Shannon Index was used to determine alpha diversity among the experimental groups, where * indicates *p* < 0.05. **(C)** The Bray-Curtis Index was applied to quantify beta diversity, and the results were displayed using ordination-based Principal Coordinate Analysis (PCoA) plots for WT (top panel) and *Trpv6^-/-^* (bottom panel). PERMANOVA testing was performed on Wild-type (F-value 1.38, R2 = 0.21, *p* < 0.12) and *Trpv6^-/-^* (F-value 1.60, R2 = 0.24, *p* < 0.09). **(D)** Supervised redundancy analysis was performed, comparing all groups in a single analysis. **(E)** Heatmaps of genus representation across experimental groups and genotypes were determined by Spearman rank correlation coefficients. Dendrograms indicate genus clustering across experimental groups.

We next analyzed the fecal microbiota to assess the effects of EtOH-feeding and CRS gut microbiome composition and the role of TRPV6 in these effects. At the phylum level, samples from all groups contained a high relative abundance of Bacteroidetes and Firmicutes, followed by lower levels of Proteobacteria (**Figure 7A**). As assessed by Shannon Index, the alpha diversity was reduced by EtOH with or without combination with CRS; CRS alone did not cause a significant change in alpha diversity (**Figure 7B**). In contrast, no alpha diversity changes were observed with any treatments in *Trpv6^-/-^* animals. Assessment using Bray-Curtis Index displayed by Principal Coordinate Analysis (PCoA) ordination plots demonstrated no significant effects on beta diversity within genotypes (**Figure 7C**). However, a supervised redundancy analysis (RDA) on all groups showed separation of wild-type EtOH and EtOH + CRS groups compared with the other groups, which clustered together except for pair-fed *Trpv6^-/-^* mice (**Figure 7D**). To better assess the specific changes driving decreased alpha diversity in wild-type EtOH alone and in combination with CRS, we performed genus-level analysis using Spearman rank correlation coefficients with heatmaps, pattern search analysis, and network analysis in wild-type and *Trpv6^-/-^* mice. The Spearman rank correlation analysis demonstrated that wild-type EtOH-fed with or without CRS contained an elevated abundance of *Bilophila, Staphylococcus, and Lactobacillus* (**Figure 7E**). Specifically, wild-type EtOH + CRS treated mice also contained a markedly higher relative abundance of *Odoribacter, Rhodococcus, Dehalobacterium, Ruminococcus*, and *unclassified Peptococcaceae, Helicobacteraceae*, and *Clostridiales.* These patterns were supported by the results of pattern search analysis (**Supplementary Figure S5A**) and network analysis (**Supplementary Figure S4A**) in wild-type mice. Similar taxa patterns were not observed in *Trpv6^-/-^* mice under the same treatment conditions (**Figure 7E**, **Figure S5B**, **Figure S6B**). Together these results suggest unique enrichment of microbes associated with pro-inflammatory and barrier dysfunction conditions in EtOH-fed with or without CRS in wild-type mice; such EtOH effects were absent in *Trpv6^-/-^* mice. These data suggest that the TRPV6 channel is involved in the EtOH-induced alteration of gut microbiota.

In a previous study, we measured plasma corticosterone levels in CRS-induced and EtOH-fed mice. Data indicated that CRS and EtOH increased plasma corticosterone; however, the level was significantly higher when CRS and EtOH feeding were combined. Therefore, in response to the reviewer’s question, we have performed a corticosterone assay in plasma in the current studies to determine the corticosterone levels in TRPV6 knockout mice. Data show similar results in both Wild-type and *Trpv6^-/-^* mice, indicating that the role of TRPV6 is downstream in glucocorticoid response (**Supplementary Figure S7**) and not involved in corticosterone release.

## Discussion

The recent finding that stress and the stress hormone corticosterone exacerbate alcohol-induced intestinal permeability and liver damage suggested that chronic stress may be a second hit in developing ALD (20, 37). However, the specific mechanism behind the stress-induced enhancement of alcohol’s effects remains obscure. A recent study indicated that the TRPV6 channel is essential in alcohol-induced intestinal epithelial TJ disruption, mucosal barrier impairment, endotoxemia, and liver injury (18). Therefore, we raised the question of whether TRPV6 contributes to stress-or corticosteroid-induced effects in the gut-liver axis and stress or corticosterone-mediated exacerbation of alcohol-induced gut permeability and systemic responses. This investigation provides evidence of TRPV6’s involvement in stress and stress-mediated potentiation of alcohol effects on the gut and liver. First, TRPV6 deficiency attenuates corticosterone-induced intestinal mucosal permeability, TJ and AJ disruption, endotoxemia, systemic inflammation, and liver damage. Second, the absence of TRPV6 blocks corticosterone-mediated enhancement of alcohol-induced gastrointestinal barrier dysfunction, endotoxemia, and liver injury. Third, TRPV6 deficiency attenuates chronic stress-induced gut barrier dysfunction and systemic effects. Fourth, the lack of TRPV6 abrogates stress-mediated exacerbation of alcohol-induced gut mucosal permeability, endotoxemia, and liver damage. Lastly, the lack of TRPV6 channel attenuates CRS and alcohol-induced dysbiosis of gut microbiota.

Systemic administration of corticosterone in mice (20) and rats (43) or local administration to the central nucleus of the amygdala in rats (44) results in increased mucosal permeability and disruption of gut barrier function. The current study confirms this effect of corticosterone on intestinal mucosal permeability in the ileum and colon. However, the mechanism of corticosterone-induced intestinal permeability is unclear. An absence of corticosterone effect on intestinal mucosal permeability in *Trpv6^-/-^* mice demonstrated that the TRPV6 channel plays a crucial role in corticosterone-induced intestinal mucosal permeability. Prevention of corticosterone-induced redistribution of TJ and AJ proteins in *Trpv6^-/-^* mice indicates that the attenuation of mucosal permeability is caused by the prevention of corticosterone-induced disruption of TJs and AJs. Barrier impairment in the intestine is expected to induce mucosal inflammation due to LPS leakage into the lamina propria. An increase in the *MCP1* mRNA in the intestinal mucosa in wild-type mice but not in *TRPV6^-/-^* mice indicated that corticosterone induces mucosal inflammation by a TRPV6-dependent mechanism.

Corticosterone treatment increased plasma LPS levels, suggesting mild endotoxemia. Elevated IL-6 and MCP1 in plasma in corticosterone-treated mice suggested that chronic corticosterone treatment induces systemic inflammation. The absence of these effects of corticosterone on plasma LPS and cytokines in *Trpv6^-/-^* mice demonstrates that TRPV6 is involved in the mechanism of corticosterone-induced endotoxemia and systemic inflammation. Endotoxemia and systemic inflammation are most likely caused by epithelial TJ breakdown and an increase in the permeability of the mucosa to LPS.

Corticosterone did not cause histopathological changes in the liver. However, corticosterone slightly elevated plasma ALT and AST activities and increased liver triglyceride levels; this confirms our recent finding of fatty liver by chronic corticosterone treatment (20). Furthermore, the current study shows that TRPV6 is involved in corticosterone-induced liver injury, as the liver injury was absent in corticosterone-treated *TRPV6^-/-^* mice. Liver injury is likely caused by corticosterone-induced gut barrier dysfunction and endotoxemia.

A recent study showed that corticosterone treatment enhances alcohol-induced gut mucosal permeability, endotoxemia, inflammatory response, and liver disease (20). The current study demonstrates that TRPV6 deficiency attenuates corticosterone-mediated promotion of alcohol-associated gut permeability, endotoxemia, and liver damage. TRPV6 increases intracellular calcium and disrupts TJ by a Src kinase-mediated mechanism (18). Corticosterone-mediated promotion of alcohol effects requires TRPV6. Precisely how corticosterone alters TRPV6 functions is currently unclear. However, it is likely that corticosterone increases the expression of TRPV6 or activates the TRPV6 channel in the intestinal epithelium. A previous study showed that the intestinal epithelial-specific knockout of the glucocorticoid receptor (GR) but not liver-specific GR knockout blocks alcohol-induced liver damage (37). Therefore, modulation of the intestinal TRPV6 channel is likely the primary mechanism of corticosterone effects on the gut and liver.

Corticosterone is rodents’ primary stress hormone elevated during chronic stress (40). The glucocorticoid cortisol (corticosterone in rodents) is the primary stress hormone, sustained elevation of which in plasma plays a crucial role in stress-induced pathophysiology. A correlation between plasma cortisol and serotonin uptake was observed in patients with chronic stress and depression (45, 46). A significant correlation between plasma cortisol and HDL cholesterol and body mass index suggests that glucocorticoids regulate critical components of cardiovascular risks (47). Exposure to stress or glucocorticoids is associated with shortened telomeres in rats (48). Corticosterone exposure impairs emotional regulation and cognitive function in mice (49). Glucocorticoid-driven NLRP3 inflammasome activation in hippocampal microglia mediates chronic stress-induced depressive-like behaviors (50). Therefore, corticosterone treatment is directly related to chronic stress pathogenesis.

To determine whether endogenously elevated corticosterone in mice produces effects similar to exogenous corticosterone, we examined the involvement of CRS on gut mucosal permeability and liver injury in wild-type and *Trpv6^-/-^* mice. Like the corticosterone effects, CRS significantly increased intestinal mucosal permeability, induced a moderate redistribution of TJ and AJ proteins in the colonic epithelium, and increased *IL-6*, *TNFα*, and *MCP1* mRNA in the colonic mucosa. An absence of these CRS effects in the gut of *Trpv6^-/-^* mice demonstrated that TRPV6 is involved in stress-induced epithelial junctional disruption, barrier dysfunction, and mucosal inflammation. In addition, the previous study showed that CRS promotes alcohol-induced gut permeability and mucosal inflammation. The current study confirms this finding and further demonstrates that the TRPV6 channel is necessary for the stress-induced enhancement of alcohol-induced gut permeability and mucosal inflammation.

The significant elevation of plasma LPS, IL-6, and MCP1 in wild-type mice indicated that CRS induces endotoxemia and systemic inflammation. The absence of such an effect in *Trpv6^-/-^* mice demonstrates that TRPV6 is necessary for these effects of chronic stress. Although CRS did not cause histopathological changes in the liver, it significantly elevated plasma ALT and AST activities and increased liver triglyceride levels indicating that CRS, like corticosterone, induced mild liver injury. Once again, TRPV6 is necessary for this effect of chronic stress on the liver. Additionally, this study confirms the previous observation that CRS potentiates alcohol-induced gut permeability, endotoxemia, and liver injury and demonstrates that TRPV6 plays an essential role in the CRS-mediated promotion of alcohol effects in the gut and liver. Although our recent study showed that the knockdown of the glucocorticoid receptor in hepatocytes failed to prevent alcohol-induced liver injury (37), it is possible that glucocorticoid receptor in other types of cells in the liver (such as Kupffer cells, sinusoidal endothelial cells) or effect of other stress hormones, such as adrenaline, affect the liver directly.

The mechanism of TRPV6 activation by EtOH or corticosterone is unclear. In a recent study, we observed that EtOH and acetaldehyde (EtOH metabolite) activated TRPV6 expressed in HEK 293 cells (18). Furthermore, this study identified H225 as a potential EtOH binding site in human TRPV6 (H185 is the corresponding residue in rat TRPV6). Mutation of H185 and R135 in the vicinity significantly reduced the EtOH-induced TRPV6 currents, suggesting that direct interaction of EtOH may regulate TRPV6 activity in the intestinal epithelium. The requirement of TRPV6 for CRS and corticosterone-induced gut permeability and liver injury suggest that corticosterone somehow activates TRPV6, the mechanism of which remains to be defined. This recent study also showed that EtOH increases intracellular calcium by a TRPV6-dependent mechanism. Therefore, the calcium-mediated downstream signaling is involved in TRPV6-dependent tight junction disruption and gut permeability. Our previous studies have demonstrated that intracellular calcium induces mitochondrial oxidative stress, which activates c-Src, leading to tyrosine phosphorylation of tight junction proteins (51).

Stress (20, 52–54) and alcohol (55–57) has been demonstrated to cause gut microbiota dysbiosis. In the current study, we examined the CRS-induced alteration of gut microbiota composition and modulation of alcohol-induced dysbiosis of microbiota by CRS in wild-type and *Trpv6^-/-^* mice. CRS failed to show a significant alteration in gut microbiota composition at the phylum level. The alpha diversity and beta diversity analyses indicated a lack of CRS effects on the microbiota diversity within and between groups. However, Spearman’s correlation and LDA analysis analyses at the genus level demonstrated that CRS caused a significant alteration in gut microbiota composition. A high abundance of genera *Dorea*, *Coprobacillus*, *Flexispira*, and *Odoribacter* is the signature feature of chronic stress under the current experimental conditions. These alterations were significantly reduced in TRPV6^-/-^ mice, demonstrating that TRPV6 plays a crucial role in CRS-induced microbial dysbiosis.

Alcohol feeding reduced microbiota diversity and caused a significant alteration of microbiota composition. CRS did not significantly alter alpha diversity or beta diversity at the phylum level. However, Spearman’s correlation and LDA analyses at the genus level showed that CRS dramatically altered alcohol-induced alteration of gut microbiota composition, with an elevated pro-inflammatory microbiota profile. A high abundance of the genera *Helicobacter*, *Bilophila*, *Odoribacter*, and *Acinetobacter* and a low abundance of *Prevotella* and *Adlercreutzia* are the signature features of the alcohol and CRS combined group. Many of these signature changes in microbiota composition were absent in *Trpv6^-/-^* mice, suggesting that TRPV6 plays a significant role in CRS and alcohol-induced gut microbiota dysbiosis. Precisely how TRPV6 plays a role in the alteration of gut microbiota is unclear. Alteration of the epithelial barrier and resulting changes in gut homeostasis may regulate microbiota composition.

An increase in the abundance of *Turicibacter*, *Bilophila*, *Staphylococcus*, *Dehalobacterium*, and *Lachnospiraceae* by EtOH and CRS was attenuated in *Trpv6^-/-^* mice. However, we cannot know whether these changes are causes or consequences of EtOH effects. For example, *Bifidobacterium* and *Lactobacillus* are increased by EtOH and CRS, and these increases are absent in *Trpv6^-/-^* mice. However, these bacteria are known to be beneficial bacteria, suggesting that the increase in these beneficial bacteria are defense mechanism caused by initial changes in microbiota composition. However, such initial changes in microbiota were absent in *Trpv6^-/-^* mice. Therefore, it is a very complex topic and needs many detailed experiments and analyses to extract information.

This finding indicates that TRPV6 plays a key role in the stress- and corticosterone-induced disruption of the tight junctions (TJ) of the intestinal epithelial cells, as well as in endotoxemia, inflammatory responses, and liver damage. TRPV6 also mediates stress and corticosterone-mediated potentiation of alcohol effects on the gut and liver. Furthermore, TRPV6 may also play a role in stress and corticosterone-induced dysbiosis of gut microbiota. These findings identify TRPV6 as a potential therapeutic target for treating stress and alcohol-associated diseases.

## Data availability statement

The data presented in the study are deposited in the NCBI BioProject repository (Submission ID: SUB12510949; BioProject ID: PRJNA918387); Individual values of data presented in different figures in the main article and the supplemental information are uploaded to FigShare (10.6084/m9.figshare.21901770).

## Ethics statement

The animal study was reviewed and approved by University of Tennessee IACUC.

## Author contributions

AM, conducted the experiments, processed, and assisted in manuscript writing. PS, conducted experiments along with ASM. CC and RR assisted AM with different assays. JP, performed the microbiota analyses and assisted in manuscript writing. RKR, designed the study plan, obtained funding, supervised the execution of studies, and wrote the manuscript. All authors contributed to the article and approved the submitted version.
