# Supplementary material for: TRPV6 deficiency attenuates stress and corticosterone-mediated exacerbation of alcohol-induced gut barrier dysfunction and systemic inflammation
Source: Front Immunol. 2023 Jan 31;14:1093584. doi: 10.3389/fimmu.2023.1093584 (PMC9929865; doi:10.3389/fimmu.2023.1093584)
Supplement: Supplementary file 1 [file DataSheet_1.pdf]

Table S1:	PCR primer sequences
**Gene**	**5′-3′ Sequence**
IL-6	Forward: TAGTCCTTCCTACCCCAATTTCC
	Reverse: TTGGTCCTTAGCCACTCCTTC
TNF-α	Forward: CCCTCACACTCAGATCATCTTCT
	Reverse: GCTACGACGTGGGCTACAG
MCP-1/CCL2	Forward: TTAAAAACCTGGATCGGAACCAA
	Reverse: GCTACGACGTGGGCTACAG
GAPDH	Forward: CTGCACCACCAACTGCTTAG
	Reverse: GGGCCATCCACAGTCTTCT

Data Availability Statement

Microbiota metagenomic sequence data are deposited to NCBI BioProject database (NCBI BioProject ID: PRJNA918387; https://www.ncbi.nlm.nih.gov/sra/PRJNA918387

Individual values of data presented in different figures in the main article and the supplemental information are deposited to FigShare Data Repository 10.6084/m9.figshare.21901770.

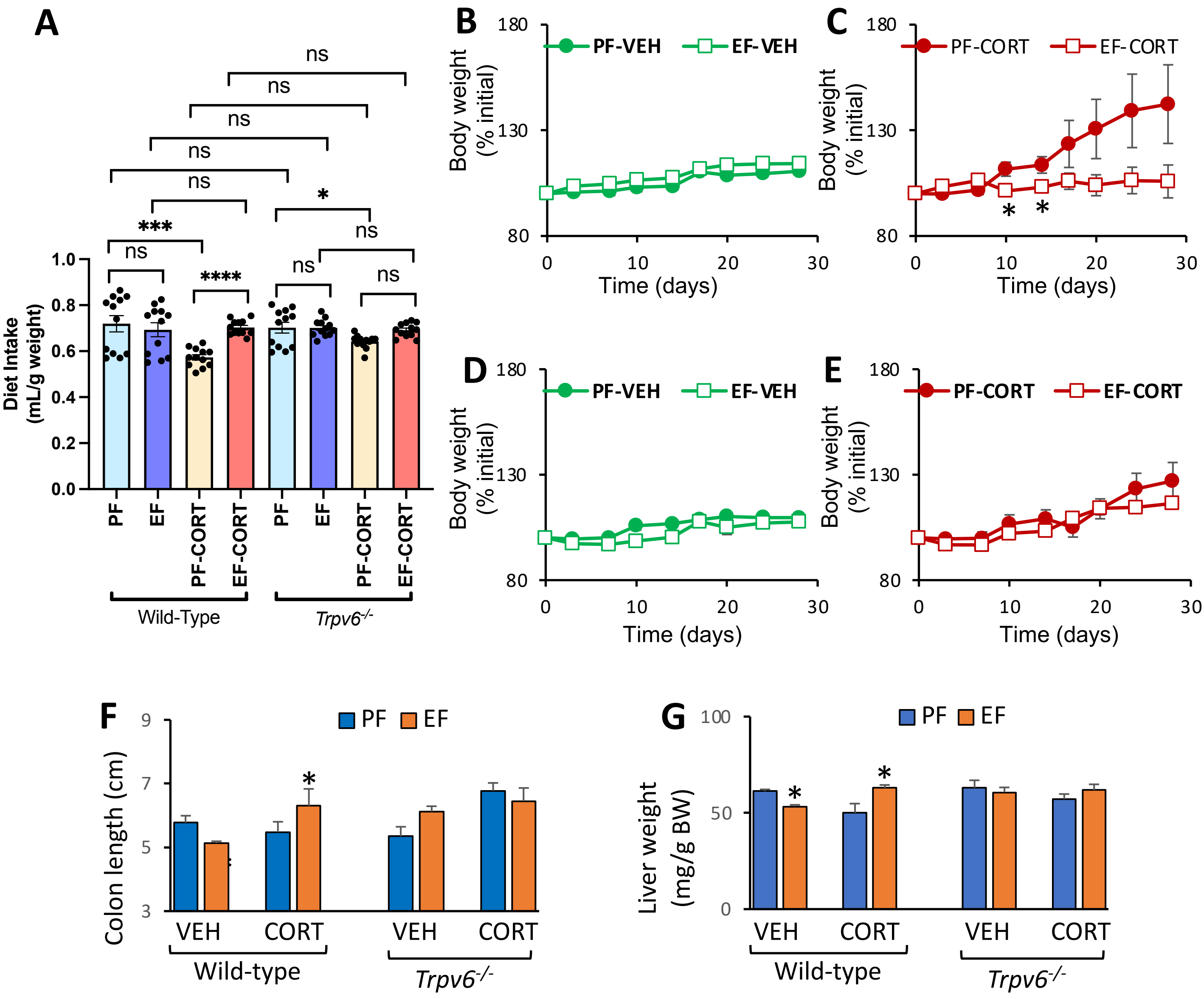

Figure S1: Effect of EtOH and corticosterone on body weight, colon length, and liver weight. Adult wild-type and *Trpv6*$^{-/-}$ mice were fed a liquid diet with EtOH (EF) or pair-fed with isocaloric maltodextrin (PF) for four weeks. In some groups, animals were injected with corticosterone (CORT) daily. Control groups received daily injection of vehicle (VEH**)**. **A:** Diet intake was measured for each pair of mice. Daily diet intake values during the last week with 6% EtOH diet are presented; * = p <0.05, *** = p <0.001 , **** = p <0.0001 for differences between indicated groups; ns = differences between the indicated groups are not statistically significant. **B-E:** Body weights as percent of 0-day values. PF vs EF comparison in VEH and CORT-treated Wild-type (B & C) and *Trpv6*$^{-/-}$ (D & E) mice. Values are Mean ± SEM (n = 4). **F:** Colon length. Values are Mean ± SEM (n = 4); * = p <0.05 for difference between corresponding PF and EF groups. **G:** Liver weights. Values are Mean ± SEM (n = 4); * = p <0.05 for difference between corresponding PF and EF groups.

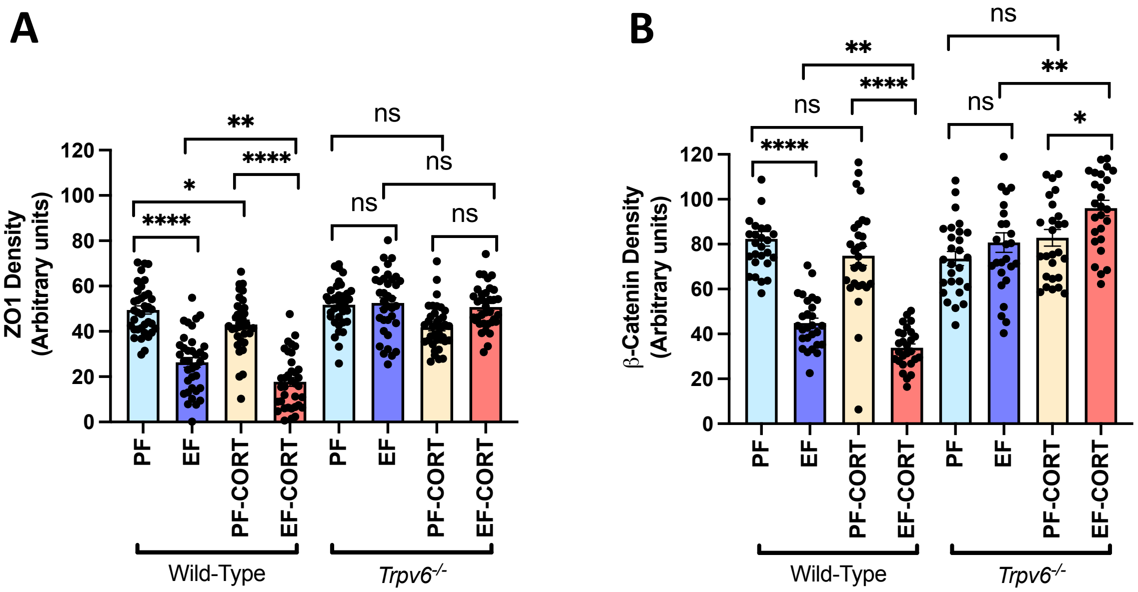

Figure S2: TRPV6 deficiency attenuates corticosterone and alcohol-induced epithelial junctional disruption. For four weeks, adult wild-type and *Trpv6*$^{-/-}$ mice were fed a liquid diet supplemented with EtOH (EF) or isocaloric maltodextrin (PF). In addition, some animal groups received daily doses of corticosterone (CORT). Other groups of animals were administered with the vehicle. Immunofluorescence labeling of colon Cryosections of colon were stained for ZO-1(A) and β-Catenin (B) by immunofluorescence method (images shown in Fig. 1). ZO-1 fluorescence densities at different locations in the epithelium were measured using Image J software. Values are fluorescence density from 8-10 locations in the epithelia from four mice per group. * = p <0.05, ** = p <0.01, and **** = p <0.0001 for difference between indicated groups..

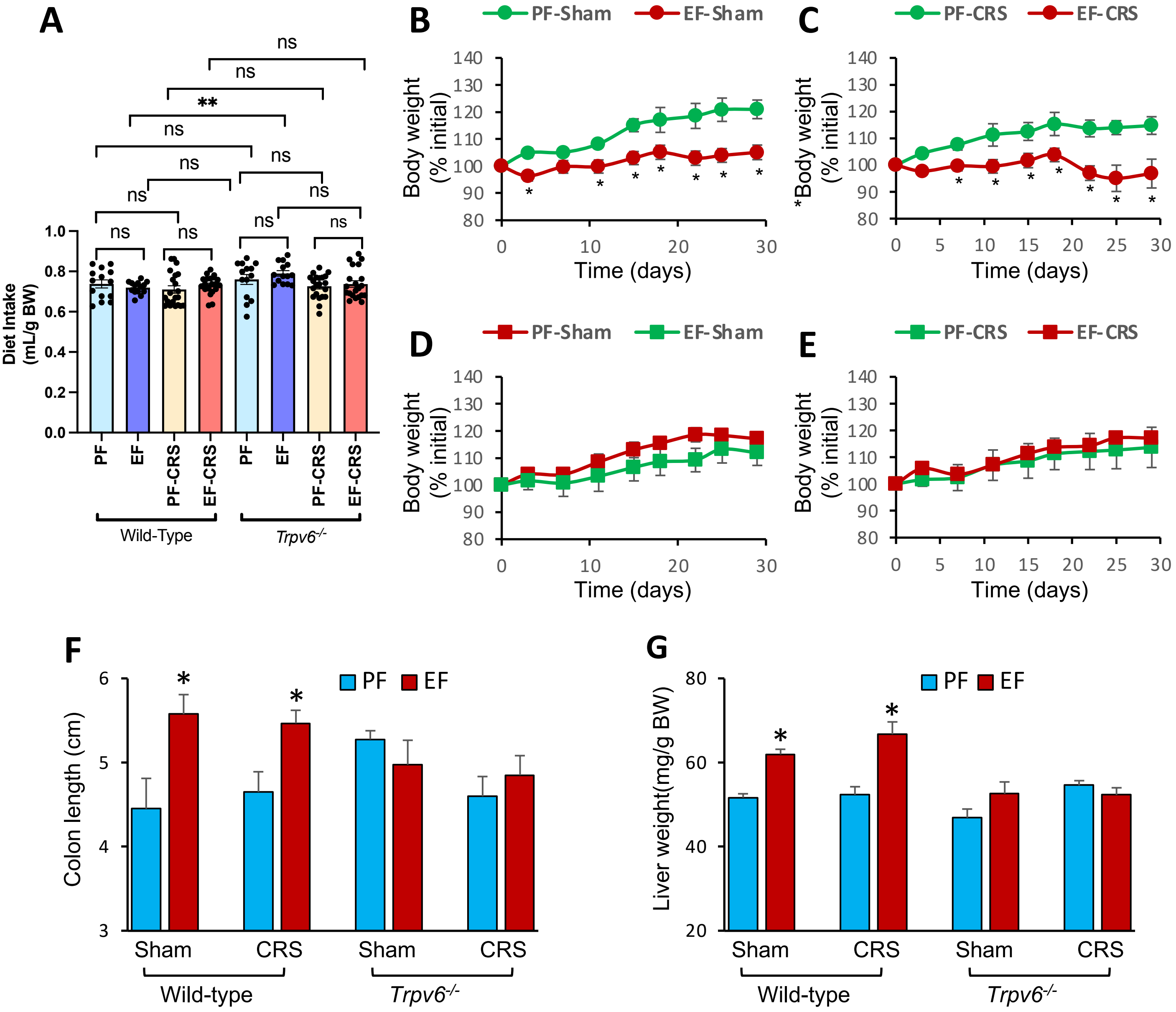

Figure S3: Effect of EtOH and CRS on body weight, colon length, and liver weight. Adult wild-type and *Trpv6*$^{-/-}$ mice were fed a liquid diet with EtOH (EF) or pair-fed with isocaloric maltodextrin (PF) for four weeks. In some groups, animals were subjected to two-hour restraint stress (CRS) daily. Non-stressed groups were deprived of food and water for 2 h daily (Sham). **A:** Diet intake was measured for each pair of mice. Daily diet intake values during the last week with 6% EtOH diet are presented; ** = p <0.01 for difference between indicated groups; ns = differences between the indicated groups are not statistically significant. **B-E:** Body weights as percent of 0-day values. PF vs EF comparison in Sham and CRS treated Wild-type (B & C) and *Trpv6*$^{-/-}$ (D & E) mice. Values are Mean ± SEM (n = 4-6); * = p <0.05 for difference between PF and EF groups. **F:** Colon length. Values are Mean ± SEM (n = 4-6); * = p <0.05 for difference between corresponding PF and EF groups. **G:** Liver weights. Values are Mean ± SEM (n = 4); * = p <0.05 for difference between corresponding PF and EF groups.

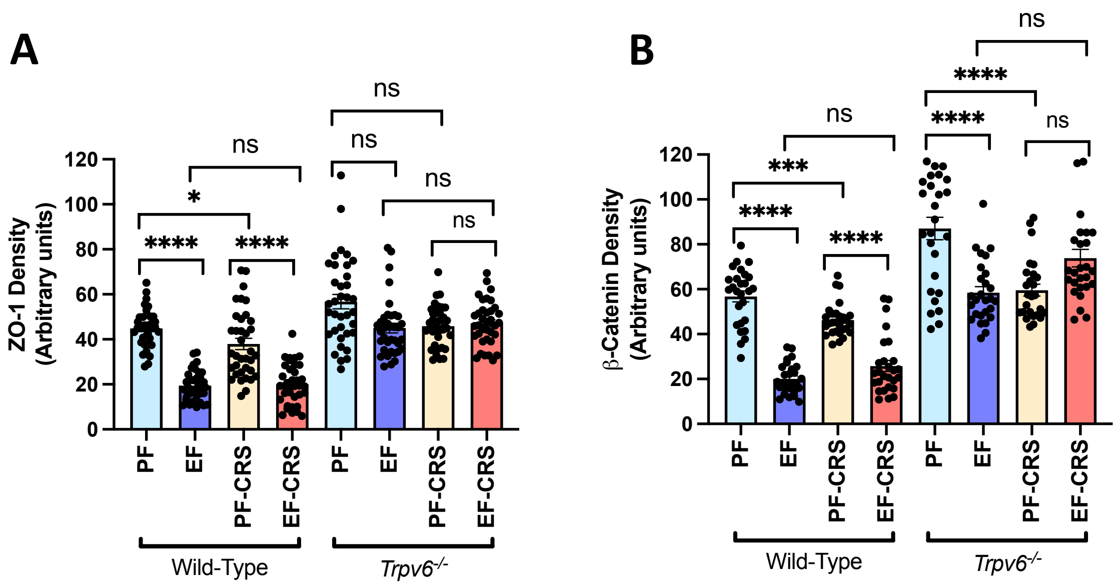

Figure S4: TRPV6 deficiency attenuates corticosterone and alcohol-induced epithelial junctional disruption. For four weeks, adult wild-type and *Trpv6*$^{-/-}$ mice were fed a liquid diet supplemented with EtOH (EF) or isocaloric maltodextrin (PF). In addition, some animal groups were subjected to CRS as described in the Methods. Other groups of animals were sham-treated. Immunofluorescence labeling of colon cryosections of colon were stained for ZO-1(A) and β-Catenin (B) by immunofluorescence method (images shown in Fig. 1). ZO-1 fluorescence densities at different locations in the epithelium were measured using Image J software. Values are fluorescence density from 8-10 locations in the epithelia from four mice per group. * = *p* <0.05, ** = *p* <0.01, and **** = *p* <0.0001 for difference between indicated groups..

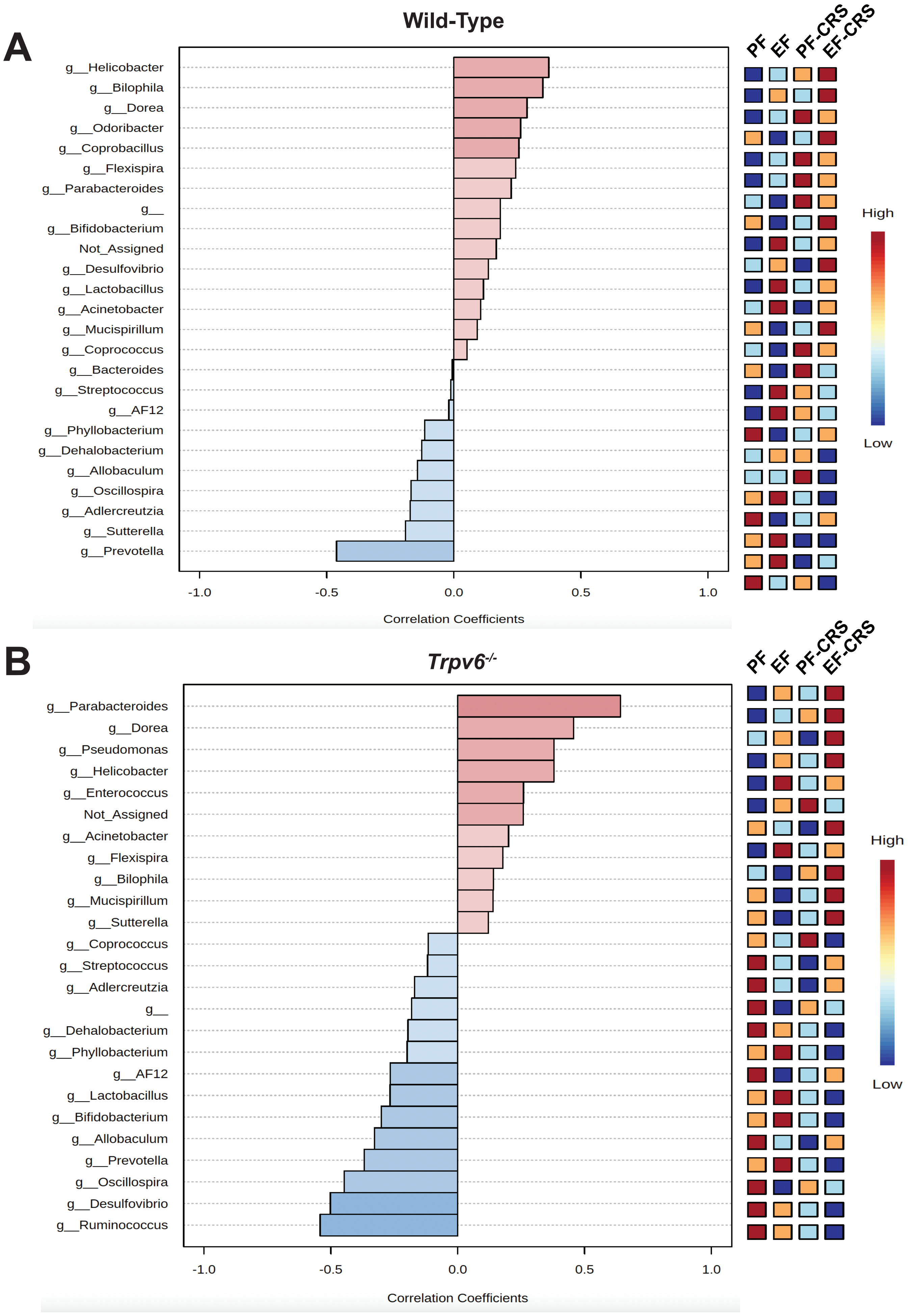
A
Wild-Type
PF
EF
PF-CRS
EF-CRS
g__Helicobacter
g__Bilophila
g__Dorea
g__Odoribacter
g__Coprobacillus
g__Flexispira
g__Parabacteroides
g__
g__Bifidobacterium
Not_Assigned
g__Desulfovibrio
g__Lactobacillus
g__Acinetobacter
g__Mucispirillum
g__Coprococcus
g__Bacteroides
g__Streptococcus
g__AF12
g__Phyllobacterium
g__Dehalobacterium
g__Allobaculum
g__Oscillospira
g__Adlercreutzia
g__Sutterella
g__Prevotella
-1.0
-0.5
0.0
0.5
1.0
Correlation Coefficients
High
Low
B
Trpv6$^{-/-}$
PF
EF
PF-CRS
EF-CRS
g__Parabacteroides
g__Dorea
g__Pseudomonas
g__Helicobacter
g__Enterococcus
Not_Assigned
g__Acinetobacter
g__Flexispira
g__Bilophila
g__Mucispirillum
g__Sutterella
g__Coprococcus
g__Streptococcus
g__Adlercreutzia
g__
g__Dehalobacterium
g__Phyllobacterium
g__AF12
g__Lactobacillus
g__Bifidobacterium
g__Allobaculum
g__Prevotella
g__Oscillospira
g__Desulfovibrio
g__Ruminococcus
-1.0
-0.5
0.0
0.5
1.0
Correlation Coefficients
High
Low

Figure S3

Figure S5: To further investigate microbiota composition, pattern search was performed on genus level identification across samples from pair-fed (PF) and EtOH-fed (EF) wild-type (A) and *Trpv6*$^{-/-}$ (B) with or without chronic restraint stress (CRS). Correlation Coefficients are displayed as positive (red) or negative (blue) bars. The log transformed relative abundance of representative taxa within each group are displayed to the right, where the legend indicates abundance within experimental groups. Positive correlations were determined in the wild-type EF+CRS group for *Ruminococcus* (0.378), *Bilophila* (0.307), and *Coprobacillus* (0.261), while AF12 (-0.379) and *Prevotella* (-0.459) were negatively correlated.

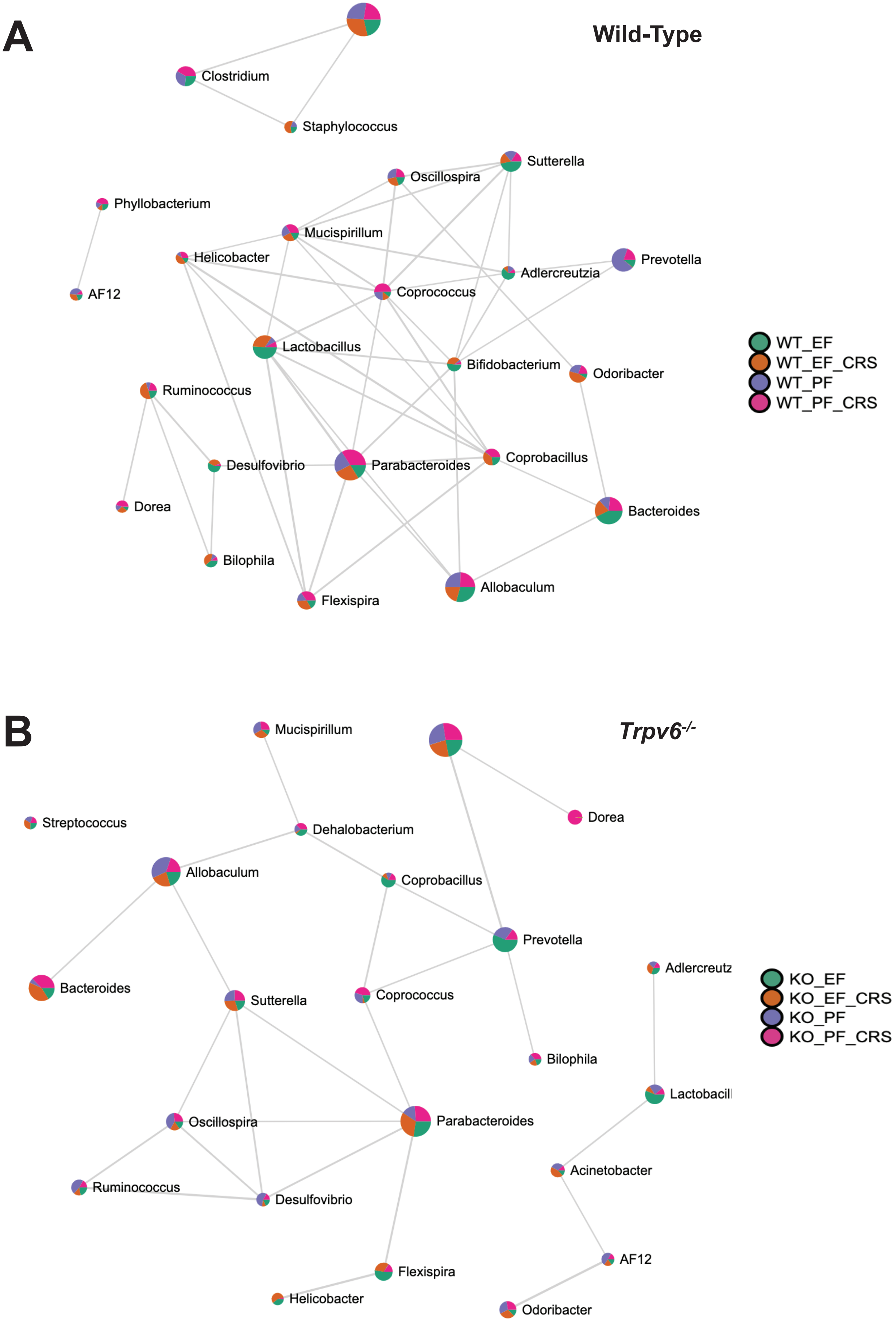
A
Wild-Type
Clostridium
Staphylococcus
Sutterella
Oscillospira
Phyllobacterium
Mucispirillum
Helicobacter
Prevotella
Adlercreutzia
Coprococcus
AF12
Lactobacillus
Bifidobacterium
Odoribacter
Ruminococcus
Desulfovibrio
Parabacteroides
Coprobacillus
Dorea
Bacteroides
Bilophila
Allobaculum
Flexispira
WT_EF
WT_EF_CRS
WT_PF
WT_PF_CRS
B
Trpv6-/-
Mucispirillum
Streptococcus
Dorea
Dehalobacterium
Allobaculum
Coprobacillus
Prevotella
Adlercreutz
Bacteroides
Sutterella
Coprococcus
Bilophila
Lactobacill
Oscillospira
Parabacteroides
Acinetobacter
Ruminococcus
Desulfovibrio
AF12
Flexispira
Helicobacter
Odoribacter
KO_EF
KO_EF_CRS
KO_PF
KO_PF_CRS

Figure S6: Network analysis of samples within pair-fed (PF) and EtOH-fed (EF) wild-type (A) and *Trpv6*$^{-/-}$ (B) with or without chronic restraint stress (CRS) were performed using SparCC correlation coefficients at the genus level. The figures demonstrate networks between genus built by the Correlation coefficients. Individual genera are displayed by nodes, where pie charts within each node indicates the representation of taxa across experimental groups, as colored by the legends. For each genotype, 100 permutations were run with a correlation threshold of 0.3.

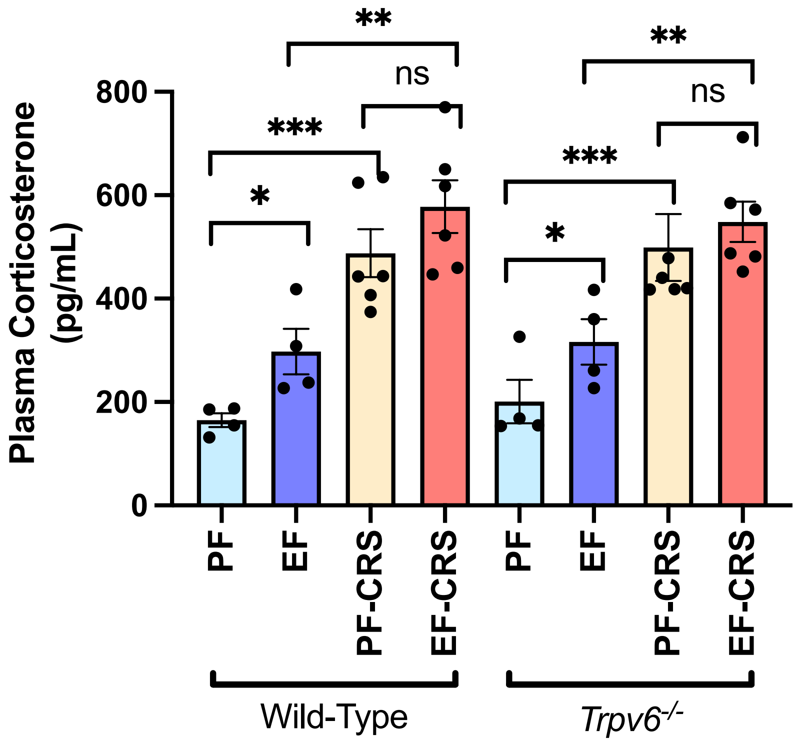

Figure S7: Effect of EtOH and CRS on plasma corticosterone. Adult wild-type and *Trpv6*$^{-/-}$ mice were fed a liquid diet with EtOH (EF) or pair-fed with isocaloric maltodextrin (PF) for four weeks. In some groups, animals were subjected to two-hour restraint stress (CRS) daily. Non-stressed groups were deprived of food and water for 2 h daily (Sham). Plasma corticosterone levels were measured. Values are Mean ± SEM (n = 4-6); * = p <0.05, * = p <0.01, * = p <0.001 for difference between indicated group pairs.
